# Genomic Instability in Multiple Myeloma: A “Non-Coding RNA” Perspective

**DOI:** 10.3390/cancers13092127

**Published:** 2021-04-28

**Authors:** Elisa Taiana, Maria Eugenia Gallo Cantafio, Vanessa Katia Favasuli, Cecilia Bandini, Giuseppe Viglietto, Roberto Piva, Antonino Neri, Nicola Amodio

**Affiliations:** 1 Department of Oncology and Hemato-Oncology, University of Milan, 20122 Milan, Italy; elisa.taiana@unimi.it (E.T.); vanessa.favasuli@unimi.it (V.K.F.); 2 Hematology, Fondazione Cà Granda IRCCS Policlinico, 20122 Milan, Italy; 3 Department of Experimental and Clinical Medicine, Magna Graecia University of Catanzaro, 88100 Catanzaro, Italy; mariaeugenia.gallocantafio@unicz.it (M.E.G.C.); viglietto@unicz.it (G.V.); 4 Department of Molecular Biotechnology and Health Sciences, University of Torino, 10126 Torino, Italy; cecilia.bandini@edu.unito.it (C.B.); roberto.piva@unito.it (R.P.); 5 Città Della Salute e della Scienza Hospital, 10126 Torino, Italy

**Keywords:** multiple myeloma, DNA repair, genomic instability, DNA damage response, base excision repair, homologous recombination

## Abstract

**Simple Summary:**

Genomic instability (GI) plays an important role in the pathobiology of multiple myeloma (MM) by promoting the acquisition of several tumor hallmarks. Molecular determinants of GI in MM are continuously emerging and will be herein discussed, with specific regard to non-coding RNAs. Targeting non-coding RNA molecules known to be involved in GI indeed provides novel routes to dampen such oncogenic mechanisms in MM.

**Abstract:**

Multiple myeloma (MM) is a complex hematological malignancy characterized by abnormal proliferation of malignant plasma cells (PCs) within a permissive bone marrow microenvironment. The pathogenesis of MM is unequivocally linked to the acquisition of genomic instability (GI), which indicates the tendency of tumor cells to accumulate a wide repertoire of genetic alterations. Such alterations can even be detected at the premalignant stages of monoclonal gammopathy of undetermined significance (MGUS) and smoldering multiple myeloma (SMM) and, overall, contribute to the acquisition of the malignant traits underlying disease progression. The molecular basis of GI remains unclear, with replication stress and deregulation of DNA damage repair pathways representing the most documented mechanisms. The discovery that non-coding RNA molecules are deeply dysregulated in MM and can target pivotal components of GI pathways has introduced a further layer of complexity to the GI scenario in this disease. In this review, we will summarize available information on the molecular determinants of GI in MM, focusing on the role of non-coding RNAs as novel means to tackle GI for therapeutic intervention.

## 1. Introduction

Multiple myeloma (MM) is an incurable malignancy of mature antibody-producing B cells, namely plasma cells (PCs), growing within a permissive bone marrow microenvironment (BMM) that triggers uncontrolled proliferation, chemo-resistance and immune evasion [1,2]. Abnormal proliferation of malignant PCs in the bone marrow (BM) frequently leads to excessive secretion of immunoglobulin (Ig) in the blood and urine, associated with organ dysfunction as hypercalcemia, renal dysfunction, anemia, and/or bone disease [3]. MM follows a multistep development process, characterized by accumulation of genomic aberrations in the malignant clone, which collectively drive the progression from precursor stages, namely monoclonal gammopathy of undetermined significance (MGUS) and smoldering multiple myeloma (SMM) to overt MM [1,2,4]. Most cancers, including MM, are characterized by GI, which defines the increased tendency of tumor cells to acquire genomic alterations ranging from simple base substitutions and small insertions or deletions to chromosome gains, loss, or rearrangements; the latter is generally referred to as chromosomal instability (CI) [5].

Several factors, either endogenous, like natural metabolic byproducts, or exogenous, such as ionizing radiation, ultraviolet (UV) radiation, and various chemical agents, can cause DNA damage, leading to genetic alterations [6,7].

The recent advent of next generation sequencing (NGS) technologies has contributed to further unravel the complex genomic landscape of MM, starting from its pre-malignant or asymptomatic phases, supporting the idea that MM onset and progression are unequivocally associated with ongoing accumulation of genomic alterations, already detectable at MGUS and SMM phases [8,9]. Various forms of CI have been detected in MM cells, which can be numerical, such as copy number alterations (CNAs) involving whole chromosomes or part of them, or structural, mainly chromosomal, rearrangements, inversions, or reassembly.

CNAs in the form of trisomies of odd numbered chromosomes (including chromosomes 3, 5, 7, 9, 11, 16, 19, and 21) are considered, along with IgH translocations, early initiating events in MM. Accordingly, MM patients are broadly classified as hyperdiploid or non-hyperdiploid: the hyperdiploid tumors are characterized by trisomies of three or more of the above-reported odd-numbered chromosomes, while the majority of non-hyperdiploid tumors display a translocation involving the IgH locus on chromosome 14 and one of the five recurrent translocation partners at chromosomes 11q13 (CCND1), 6p21 (CCND3), 4p16 (FGFR3 and NSD2), or 16q23 (MAF) [10,11]. Overall, the above-mentioned chromosomal abnormalities can alreadybe detected in PCs of MGUS and SMM patients; however, additional genetic alterations are required for progression towards a clinically active disease [8,12]. These include translocations, deletions, and chromosome gains, involving genes such as MYC, KRAS, NRAS, and TP53, some of which are implicated in the DNA damage response (DDR). Secondary events are generally detected in the late stage of the disease [12,13].

In this review, we will outline available information on the most well-characterized molecular mechanisms underlying GI in MM, mostly focusing on the recent evidence that deranged non-coding RNA-based networks may contribute to ongoing GI and represent potential targets for therapeutic intervention.

## 2. Molecular Basis of GI in MM

Thus far, the most documented mechanisms of GI in MM involve abnormal DNA repair processes and defective replicative stress, though novel mechanisms are emerging that need to be fully elucidated [8,14,15].

### 2.1. Alterations of DNA Repair Pathawys

#### 2.1.1. Deregulated Expression of DNA Repair Genes

DNA repair is critical to target extrinsic or intrinsic DNA damage, ensuring regulated gene transcription and DNA replication. To ensure cell survival, specific protein networks interact and collaborate to detect and repair DNA damage through a process collectively referred to as DDR. Specific DNA repair pathways are required to repair the different type of DNA lesions, mostly represented by DNA single-strand breaks (SSBs), DNA double-strand breaks (DSBs), and interstrand crosslinks (ICLs). Deregulation of these pathways are implicated in the onset and maintenance of human cancers, including MM.

The repair of SSB DNA is mediated by several pathways, such as base excision repair (BER), nucleotide excision repair (NER), or mismatch repair (MMR), while non-homologous end joining (NHEJ) and homologous recombination (HR) pathways can repair DNA DSBs. The Fanconi Anemia (FA) pathway is instead responsible for ICL repair (see more specialized reviews [16,17,18] for further details). Alterations or deregulation in crucial genes or proteins involved in the different DDR processes in MM patients were reported by independent research groups and are highlighted and summarized in Table 1.

With regard to the BER system, a single nucleotide polymorphism (SNP) within the OGG1 gene was found to be associated with the occurrence and increased risk of disease progression in MM patients [19,20]. Furthermore, polymorphisms in two other BER pathway genes, APE1 or MUTYH, represent independent prognostic factors significantly associated with the shorter survival of MM patients [20].

The NER pathway was reported to be activated in MM, and it was demonstrated that its inhibition induces a chemo-sensitizing effect to alkylating agent treatment. In line with this evidence, Botta et al. demonstrated that high RAD23B, XAB2, and POLD3 expression is associated with poor prognosis of MM patients [21]. Furthermore, it was reported that specific targeting of the major NER gene ERCC3, overexpressed in MM, could be particularly efficient for MM treatment [22].

Deregulation of the MMR pathway was also reported in MM, highlighting an increased frequency of defects in this pathway, associated with more aggressive stages of the disease, and suggesting their contribution in disease progression. Furthermore, the same group highlighted a strong association between reduced DDR and the aberrant expression of at least one MMR protein, including MLH1, MSH2, and PMS1 [23], resulting in a higher mutation rate, particularly within microsatellite DNA regions, leading to GI.

DNA DSBs can be caused by exogenous agents, such as ionizing radiation or chemicals, or endogenously by ROS, replication of single-strand breaks, or replication stress. Two main DDR pathways, NHEJ and HR, are involved in DNA DSBs. NHEJ occurs during any phase of the cell cycle; although this pathway is very fast to seal DSBs, it could lead to loss or changes of nucleotides, and, therefore, it represents an error-prone repair process [24]. On the contrary, HR occurs in the late S- or G2-phase of the cell cycle, when cells have an undamaged sister chromatid to be used as a homologous DNA template to fix DSBs correctly.

Elevated activity of the NHEJ repair pathway has been observed in MM, together with its significant association with poor overall survival [25]. In line with this observation, LIG3 mRNA expression progressively increased in PCs from healthy donors to MM and plasma cell leukemia (PCL) patients and significantly correlated with shorter overall survival (OS) and event-free survival (EFS) [26]. Furthermore, Herrero et al. showed that DNA-PKcs, XRCC4, and Artemis were clearly upregulated in MM cell lines compared to control cells [27]. Interestingly, higher expression of XRCC4 has previously been reported in tumor samples isolated from patients with MM [28]. The upregulation of these NHEJ proteins is likely to contribute to the increased repair efficiency observed in MM cells.

Moreover, association between polymorphisms or deregulated expression of the XRCC5 (encoding KU80), XRCC6 (encoding KU70), LIG4 (encoding DNA ligase 4), Artemis, or XRCC4 genes and the potential risk of developing MM has been described [28,29,30].

Kumar et al. highlighted that two BER proteins, belonging to the category of apurinic/apyrimidinic (AP) nucleases, APEX1 and APEX2, contribute to regulating the HR process in MM, suggesting a potential use of AP nuclease inhibitors in combination with the alkylating agent melphalan to induce a synergistic cytotoxic effect in MM [31]. In such a context, Shammas et al. reported the upregulation of both APEX1 and APEX2 genes in MM cell lines and patient samples, with respect to normal PCs, demonstrating an increased HR activity in primary MM cells and MM cell lines, compared with healthy PCs based on the evidence of increased expression of mRNA and protein levels of RAD50 and RAD51 [32]. The induction of HR activity leads to a significant increase in the number of new mutations over time, as well as development of drug resistance in MM cells, suggesting that dysregulated HR activity in MM could be considered a potential therapeutic target [32].

An important role in the DDR to DSBs was demonstrated by Shah et al. for the MMSET gene, encoding a histone methyltransferase overexpressed in t(4;14) MM patients. The authors showed that MMSET is required for efficient NHEJ and HR processes; importantly, MMSET loss was associated with the down regulation of several DNA repair proteins, as well as the decreased recruitment of DNA repair proteins to DNA DSBs sites. By using t(4;14 MM cell lines with constitutive expression of MMSET, the authors found that these cells had increased DNA damage repair activity at baseline. Specifically, upon treatment with DNA-damaging agents, these cells repaired DNA damage at an enhanced rate and continued to proliferate, whereas those negative for the t(4;14) accumulated DNA damage and entered cell cycle arrest. By means of an in vivo experiment, the authors demonstrated that MMSET depletion had a chemo-sensitizing effect [33].

ICLs are covalent links between two opposite DNA strands, induced by endogenous metabolites and exogenous chemicals, such as alkylating agents. The FA pathway detects ICLs and repairs ICL lesions in co-operation with NER and HR pathways and greatly influences drug response [34]. In this regard, it was reported that many FA/BRCA genes are overexpressed and causative of drug resistance in melphalan-resistant MM cell lines [35].

#### 2.1.2. Mutations in DNA Repair Genes

Mutations in DNA repair genes, mainly involving tumor suppressor genes, lead to increased mutation frequency and GI in cancer [36].

In agreement with this, massive sequencing of paired tumor/normal samples obtained from 203 MM patients allowed Lohr et al. to confirm previous findings of a significant fraction of MM patients carrying mutations in the TP53 gene [37,38]. Furthermore, in 2015, Cifola et al. performed a whole-exome sequencing analysis of a prospective series of 12 primary PCL (pPCL) cases, highlighting TP53 as the most recurrently disrupted gene [39]. Furthermore, among 14 genes with a potential driver role in pPCL, the authors identified KIF2B, known to play an important role in genome stability by regulation of microtubule attachment to chromosomes during mitosis.

Moreover, Walker et al. performed whole-exome sequencing for 463 MM patients, reporting alterations in crucial genes of the HR pathway, such as TP53, ATM, and ATR, highlighting the association of these mutations with inferior patient survival [40]. In 2018, Pawlyn et al. confirmed that ATM was frequently mutated in MM patients, together with the BRCA2 gene, suggesting the importance of the identification of MM patients with inherent BRCAness, which may be more likely to respond to single agent PARP inhibition [41].

APOBEC DNA cytosine deaminases could be considered potential genomic mutators in various cancers. APOBEC is involved in antiviral defense by restricting retrovirus propagation and transposons mobility through the introduction of DNA lesions. The ability of APOBEC enzymes to use ssDNA as a substrate has been reported, which inevitably occurs during transcription and replication of DNA, leading to oncogenic mutations.

Kanu et al. reported a strong association between APOBEC-induced mutagenesis and replicative stress by demonstrating that APOBEC3B activation can be counteracted through alleviation of replication stress with nucleoside supplementation. Furthermore, they demonstrated that, in the condition of replicative stress, prolonged exposure of ssDNA can increase DNA susceptibility to APOBEC-induced mutagenesis [42].

In line with these findings, this susceptibly was exacerbated upon APOBEC hyperactivation [43]. Accumulation of APOBEC signature mutations increases significantly in refractory MM tumors and extramedullary forms [44] and is associated with poor prognosis [14,15]. In fact, Walker et al. reported the presence of an APOBEC mutational signature in MM samples linked to the translocation-mediated deregulation of MAF and MAFB, a known poor prognostic factor, while the loss of MAF or MAFB expression results in decreased APOBEC3B and APOBEC4 expression indicated a transcriptional control [15].

NGS analyses have led to the identification of more than 17 mutational signatures in MM genomes, including both single-base substitution mutational signatures [44,45,46] and de novo chromosomal structural rearrangements [8,47], extending or confirming previous findings on elevated GI and CI in MM PCs.

**Table 1:** Crucial genes of the DNA damage response found deregulated in MM.

DNA Repair Pathway	Alterated Gene in MM	Type of Alteration	Resulting Effect	Reference
BER	OGG1	SNP	Low BER activity, increased risk of disease progression in MM patients	[19,20]
	MUTYH	SNP	Shorter survival of MM patients	[20]
	APE1	SNP	Shorter survival of MM patients	[20]
NER	RAD23B	Overexpression	Poor prognosis of MM patients	[21]
	XAB2	Overexpression		[21]
	POLD3	Overexpression		[21]
	ERCC3	Overexpression	Activation of NER pathway	[22]
MMR	MLH1	Aberrant expression/deficiencies in proteins	Reduced functionality of MMR and higher mutation rate	[23]
	MSH2			[23]
	PMS1			[23]
HR	APEX1	Upregulation	Increased HR activity, increase in the number of new mutations, and development of drug resistance in MM	[32]
	APEX2	Upregulation		[32]
	RAD50	Upregulation		[32]
	RAD51	Upregulation		[32]
	TP53	Mutation	Inferior MM patient survival	[37,38,39,40]
	ATM	Mutation		[40,41]
	ATR	Mutation		[40]
	BRCA2	Mutation		[41]
	MMSET	Overexpression	Overexpression of several DNA repair proteins	[33]
NHEJ/ALT-NHJ	LIG3	Upregulation	Shorter OS and EFS	[26]
	DNA-PKcs	Upregulation	Elevated NHEJ activity	[27]
	XRCC4	SNP/Upregulation	Potential risk of developing MM/Elevated NHEJ activity	[27,28,29,30]
	XRCC5	SNP/deregulated expression	Potential risk of developing MM	[28,29,30]
	XRCC6	SNP/deregulated expression		[28,29,30]
	LIG4	SNP/deregulated expression		[28,29,30]
	Artemis	SNP/Upregulation	Potential risk of developing MM/Elevated NHEJ activity	[27,28,29,30]
	MMSET	Overexpression	Overexpression of several DNA repair proteins	[33]

### 2.2. Replication Stress

To ensure the integrity of the genome during replication, high-fidelity DNA replication proceeds via bidirectional replication forks (RF). Replication stress (RS), which is acknowledged as a relevant source of GI in MM, can be defined as the transient slowing or stalling of the replication forks. RS is mostly dependent on the activation of oncogenes and/or the inactivation of tumor suppressor genes, which can directly induce RS by stalling and collapsing RFs or indirectly induce RS by enforcing premature transition towards the S phase [48]. It is noteworthy that the processing and repair of single-ended DSBs emerging from collapsed RFs are highly error-prone and generate mutations and complex genomic rearrangements [49]. RS occurs early in MGUS and gradually increases during MM progression [50,51]. Ongoing RS and associated DNA damage significantly enhance the mutation rate via activation of low fidelity DNA repair pathways, leading to acquisition of GI. In line with this assumption, it has been found that the HR machinery mediates the response to RS by restarting stalled RFs and repairing single-ended DSBs that result from collapsed RFs through error-prone mechanisms, causing complex genomic rearrangements that drive tumorigenesis. Break-induced replication, a type of HR that predominantly repairs single-ended DSBs resulting from altered RFs, works by engaging RAD51 to mediate strand invasion of a homologous DNA to restart stalled RFs; the invaded strand may be released by branch migration, and the newly extended double-stranded DNA end repaired by microhomology-mediated end-joining leaves a tandem duplication [52]. This phenomenon, known as microhomology-mediated break-induced replication (MMBIR), fills the DNA gap by NHEJ. Moreover, MM cells display elevated expression of RAD51 and a high frequency of spontaneous RAD51-mediated HR events, sustaining GI [32]. Interestingly, pharmacologic targeting of RAD51 increased the frequency of spontaneous DSBs, leading to MM cell apoptosis [53]. RS also cooperates with transcriptional stress to the ongoing GI observed in MM, as supported by the finding that RS mostly occurs in highly transcribed PC-specific genes [8,47].

### 2.3. Newly Identified GI Mechanisms

Novel mechanisms involved in GI and CIN are continuously emerging. Chromothripsis and chromoplexy are recently discovered GI mechanisms involving random breakage and fusion of DNA. Chromothripsis occurs early in myelomagenesis as a single catastrophe event, typically involving hundreds of locally clustered rearrangements, affecting only one or a few chromosomes as a consequence of NHEJ mechanisms [54]. Conversely, chromoplexy is a late event in MM, resulting from DSBs in several chromosomes which occur simultaneously and are rejoined incorrectly [55].

## 3. Non-Coding RNA Involvement in MM GI

**Figure 1:**
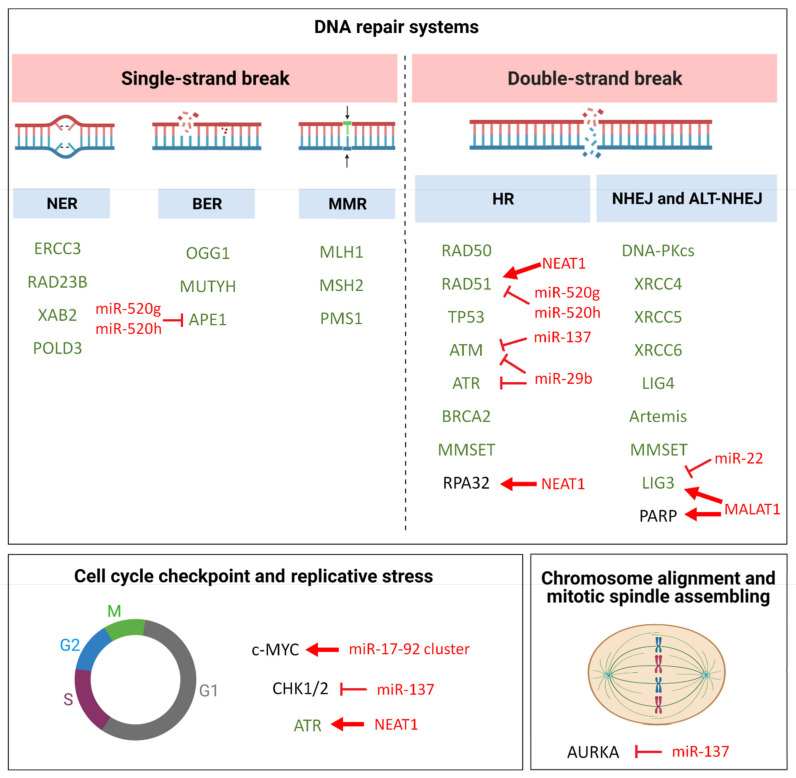
Schematic diagram illustrating pathways involved in GI molecular mechanisms in MM. Genes with known aberrant expression or function in MM are reported in green and discussed in the main text; miRNA and lncRNAs targeting crucial players of these pathways in MM are reported in red, along with their putative/predicted molecular effect. Red bar-headed arrows indicate inhibiting effects, while red arrows indicate activating effects.

The identification and characterization of the non-coding genome have added a further layer of complexity to the regulatory mechanisms underlying GI. In this section, we will provide an overview of the most relevant classes of non-coding RNAs (ncRNAs) involved in GI in MM; their mechanisms of action and their role as tumor suppressor or oncogenic ncRNAs will be highlighted (see Table 2 and Figure 1).

### 3.1. miRNAs

MicroRNAs (miRNAs) are short ncRNAs of ~22 nucleotides (nt) in length that post-transcriptionally regulate gene expression upon binding to a target mRNA sequence through its 5′ end (known as a “seed sequence”). miRNAs are estimated to regulate the translation of more than 60% of protein-coding genes [56] and are involved in almost every cellular process.

Biogenesis of miRNAs takes place through a multi-step process that begins with transcription, by RNA polimerase II, of a primary transcript (pri-miRNA), followed by its cleavage by the RNA endonuclease Drosha. Such a cleavage leads to a 70–100 bp pre-miRNA, which is translocated in the cytoplasm by exportin 5; here, the endonuclease Dicer cleaves the pre-miRNA, leading to 20–22 bp miRNA/miRNA* duplexes. Thereafter, the mature miRNA strand is loaded onto the RNA-induced silencing complex (RISC) to induce translational repression or degradation of mRNAs, following a partial or complementary binding to the 3′UTR (untranslated region), respectively [57]. Notably, miRNAs are able to target hundreds of mRNAs, leading to a complex and combinatorial regulation of multiple pathways. On this basis, it is reasonable that alterations in the expression of miRNAs could underlie the pathogenesis of various diseases, including MM [58,59,60].

#### 3.1.1. miR-29b

miR-29b is an established tumor suppressor miRNA in MM, which targets epigenetic regulators, such as DNMT3A/B [61] and HDAC4 [62]. In the context of validated MM preclinical models, it has been shown that miR-29b overexpression triggers anti-MM activity by promoting cell cycle arrest and apoptosis [63,64]; moreover, miR-29b enforcement reduced the formation of mature human osteoclasts from their precursors, suggesting an inhibitory role on the development of MM-related bone disease [65]. Importantly, miR-29b was found to be downregulated in MM-associated dendritic cells (DCs) as compared to normal mature DCs. Enforcement of miR-29b in DCs co-cultured with MM cells counteracted pro-inflammatory pathways, including STAT3, NF-κB, and various cytokine/chemokine signaling networks, and antagonized DC-induced polarization of T helper lymphocytes into Th17 cells [66,67]. An inflammatory BMM, including DCs, has been reported to promote GI in MM, which in turn promotes the arising of mutations responsible for tumor progression, drug resistance, and immune escape [68]. Of note, after co-culture with MM DCs, accumulation of DSBs was observed in MM plasma cells, which was reduced by enforcing the expression of miR-29b in DCs; interestingly, in the latter cells, a decrease was observed in the phosphorylation of ATM, ATR, their downstream molecules CHK1 and CHK2, and H2AX, as compared with healthy DCs [66].

#### 3.1.2. miR-22

Hyperactivation of DNA ligase 3 (LIG3) is relevant for GI and survival of MM cells. LIG3 mRNA was found to be highly expressed in MM PCs, correlated with a worse outcome in MM patients and increased during progression towards extramedullary disease. LIG3 knockdown strongly increased DNA damage of MM cells, as determined by an increase in H2AX expression, and inhibited MM cell growth in vitro and in vivo, supporting the key role played by LIG3 in ALT-NHEJ, a highly error prone DNA repair pathway involved in GI [26]. Moreover, miR-22 inversely correlated with LIG3 mRNA levels in MM patients; indeed, LIG3 mRNA was validated as a direct miR-22 target in MM, which acted as a tumor suppressor miRNA. Of note, ectopic expression of miR-22 inhibited LIG3-mediated nuclear and mitochondrial DNA repair, and increased unrepaired DNA damage, which ultimately led to apoptosis of MM cells. Finally, upregulation of LIG3 promoted bortezomib resistance, and LIG3 downregulation or miR-22 overexpression were highly cytotoxic and partially restored drug sensitivity in bortezomib-resistant MM cells. On this basis, it was postulated that MM cells, in order to survive ongoing endogenous or drug-mediated DNA damage, redirect the DNA repair machinery towards LIG3-driven DNA repair, which repairs nuclear and mitochondrial DNA, allowing the acquisition of new genetic changes relevant for disease progression and drug resistance [26].

#### 3.1.3. miR-137

CI, i.e., the inability to maintain correct chromosome complement after mitosis, is common in MM patients, known to exhibit a wide range of genomic abnormalities, such as the t(4;14) translocation, MAF translocations, gain of chromosome 1q, and deletion of chromosome 17p [12].

miR-137 is a tumor suppressor miRNA in several tumors [69,70]. It is located in the frequently deleted region of chromosome 1p22. In MM, miR-137 acts as a tumor suppressor and its low expression is associated with the 1p22 deletion [71]. Qin et al. reported low expression levels of miR-137 in MM patients, which correlated with shorter progression free survival and overall survival compared with high miR-137-expressing patients. In MM PCs, silencing of miR-137 was due to increased promoter methylation. Of note, miR-137 overexpression was able to decrease the incidence of chromosome 1q21 gains and 1p22.2, 14q, and 17p13 deletions, which are generally present in patients at advanced stages. The authors attributed these effects to the targeting of Aurora Kinase A (AURKA), a serine threonine kinase playing an essential role in chromosome alignment, centrosomal amplification, and mitotic spindle formation. AURKA phosphorylates CHK1/2 and other DNA repair proteins thus dysregulate the DDR response. miR-137-induced AURKA downregulation led to decreased levels of phosphorylated ATM/CHK2 and phosphorylated BRCA1, enhancing p53 and p21 expression, which resulted in in vitro and in vivo anti-MM activity, as well as increased bortezomib sensitivity [72].

#### 3.1.4. miR-520g and -520h

By screening isogenic bortezomib sensitive and resistant MM cell lines, Yuan et al. demonstrated differential miRNA profiling [73]. In particular, miR-520g and miR-520h, two miRNAs located on the human chromosome 19 and belonging to the miR-515 family [74], were downregulated in drug resistant cell lines. In MM, bortezomib significantly reduced the expression of RAD51, an HR-related protein, indicating the anti-DNA repair function of the drug [75]. APE1 is an important BER DNA repair protein that contributes to HR dysregulation through transcription control of RAD51, as well as its ability to induce DNA breaks [76]. APE1 was found to be upregulated in bortezomib-resistant MM cell lines and was validated as a direct target of miR-520g and miR-520h, whose overexpression alleviated drug resistance of MM cells; such effects were rescued by APE1 overexpression. Importantly, combined miR-520g and miR-520h overexpression reduced the growth of bortezomib-resistant MM xenograft, underscoring the potential of miR-520g/h replacement strategies for the treatment of MM, even in the refractory setting [73].

#### 3.1.5. miR-17-92 Cluster

Botta et al. demonstrated the upregulation of genes belonging to NER in MM cells as compared to normal PCs. Specifically, 7 out of 31 genes involved in the NER system were significantly deregulated in 4 out of 5 datasets analyzed. Among them, high RAD23B, XAB2, and POLD3 expression was associated with poor prognosis, whereas a higher expression of XPA was associated with better survival [21].

Based on this evidence, the authors investigated the anti-MM activity of trabectedin (Ecteinascidin 743), whose mechanism of action relies on NER system expression [77]. This drug binds to the minor groove of DNA and traps the NER machinery as it attempts to repair DNA, leading to the generation of lethal DNA double strand breaks. Trabectedin triggered potent anti-myeloma activity in cell lines and primary cells at nanomolar concentrations, both in conventional 2D and advanced 3D models, eliciting both direct cytotoxicity on MM cells and also activating the innate immune response against MM through the upregulation of NKG2D ligands MICA/B and ULBP1. The relevance of NK response in MM pathogenesis has been deeply investigated in the past, and a downregulation of surface expression of MICA on malignant PCs or a decline in NK-dependent immune-surveillance was observed when MGUS progresses towards active MM [78].

Mechanistically, trabectedin increased the expression level of the MICA/B-positive regulator E2F1 and reduced the expression of the negative regulators IRF4 and IKZF1. Furthermore, taking into account that reprogramming of the immune response requires rapid changes at both the transcriptional and post-transcriptional levels, the authors hypothesized a role for miRNAs in finely tuning this regulatory network. By using miRNA target prediction tools, the miR-17 family was identified as the most relevant in MM biology, predicted to target, at the same time, MICA, MICB, and E2F1.

MiR-17-92 is an oncogenic cluster of miRNAs, encoded by *MIR17HG* at 13q31.3 [79]. Morelli et al. identified a MYC/miR-17-92 feed-forward loop that maintained the expression of BIM, and likely other co-regulated genes, at homeostatic levels, allowing MM cells to proliferate; moreover, they showed that MIR17PTi, a specific LNA gapmeR inhibitor selectively targeting the MIR17HG primary transcript, was able to disrupt the MYC/miR-17-92 loop and trigger apoptosis by inducing MYC-dependent synthetic lethality. MIR17PTi antagonized in vivo growth of human MM cells as a single agent, as demonstrated in four different and clinically relevant murine models, including those refractory to conventional anti-MM agents and orthotopic systems, in which MM cells grow within a human BM milieu [80]. Trabectedin downregulated miR-17 and miR-20a, and miR-17-92 stable overexpression reduced trabectedin-dependent upregulation of NKG2D ligands, confirming a role for the miR-17-92 cluster in mediating, at least partially, trabectedin effects in MM [21].

### 3.2. lncRNAs

Long non-coding RNAs, which are acknowledged as transcripts of more than 200 bp in length, have been involved in different biological processes, such as cell differentiation [81], epigenetic regulation of gene expression, and modulation of nuclear architecture, X-inactivation, and gene imprinting [82,83,84]. According to their genomic location. with respect to the nearest protein-coding genes, lncRNAs can be classified as: (i) long intergenic non-coding RNAs (lincRNAs), which do not lie close to protein-coding genes; (ii) sense lncRNAs, which are on the same strand of protein-coding genes and are transcribed in the same direction; (iii) antisense lncRNAs, which lie on the opposite strand of protein-coding genes with which they overlap; (iv) intronic antisense lncRNAs and bidirectional lncRNAs, located on the other strand, with respect to protein-coding genes and transcribed in the opposite direction [85,86,87].

Different classes of lncRNAs are transcribed from several DNA elements, such as enhancers (eRNAs), promoters, and intergenic regions (lincRNAs) in eukaryotic cells [88]. LncRNAs are cell-type specific and tissue-specific. Through their interaction with RNA, DNA, or proteins, they exert their functions through distinct molecular mechanisms [89]. LncRNAs are involved in both activation and inhibition of gene expression, playing a role as regulators of the combinatorial actions of transcription factors [90]. Additionally, it has been found that lncRNAs may act as a decoy by binding transcription factors or proteins and thereby precluding their action on target DNA. For example, the lncRNA MALAT1 binds nuclear splicing factors into nuclear speckles and also functions as a sponge to miRNAs [91,92]; NEAT1 can mediate changes in the cell transcriptome by negative regulation of effector molecules [93]. Another established function of lncRNAs is to recruit and guide molecules for chromatin-modifying complexes to the target genes [89]. Finally, lncRNAs can bind multiple proteins and facilitate the formation of extensive networks of ribonucleoprotein (RNP) complexes with numerous chromatin regulators acting as scaffolds. The prototypical example of the lncRNA scaffold is the telomerase RNA component TERC, which assembles the telomerase complex for the maintenance of the GI [94].

#### 3.2.1. MALAT1

Metastasis-associated lung adenocarcinoma transcript 1 (MALAT1) is a conserved lncRNA, acting as oncogene in a wide variety of solid and hematological malignancies. In MM, MALAT1 was found to be upregulated during the progression from intramedullary to extramedullary disease. Mechanistically, MALAT1 promotes cell survival by regulating the expression and activity of the proteasome machinery. Indeed, MALAT1 represents a druggable target in MM, as demonstrated by the significant anti-tumor activity promoted by selective LNA gapmeRs targeting MALAT1 in vitro and in vivo in NOD-SCID mice bearing MM xenografts [60,85,95]. Hu et al. demonstrated that MALAT1 overexpression in MM may trigger a NHEJ DNA repair pathway to induce secondary chromosome changes, likely promoting disease progression and drug resistance. MALAT1 was demonstrated to act as a molecular scaffold in the formation of PARP1-LIG3 complexes that recognize the DSBs on DNA and activate the A-NHEJ DNA repair in MM cells. Importantly, MALAT1 inhibition by antisense oligonucleotides effectively synergized with both PARP inhibitors and proteasome inhibitors [96].

#### 3.2.2. NEAT1

Nuclear paraspeckle assembly transcript 1 (NEAT1) is a functionally conserved lncRNA, abundantly expressed in a variety of mammalian cell types and found to be deregulated in various types of cancers; it has been implicated in the regulation of apoptotic cell death, cell growth, proliferation, invasion, and metastasis [97,98].

NEAT1 represents an indispensable structural component of nuclear paraspeckles (PSs), a class of subnuclear bodies found in the interchromatin space of mammalian cells [99], potentially involved in the nuclear sequestration of specific RNAs or proteins and stress responses [100].

Increasing evidence highlighted the crucial role of NEAT1 and essential structural proteins of PSs (PSPs) in the direct and indirect regulation of the DDR system [101].

Importantly, NEAT1 was identified as a p53 target [102,103]. In line with this, Adriaens et al. demonstrated that activation of p53 increased PS formation in mice and human cells. Furthermore, the same group found that NEAT1 silencing in mice prevented PS formation, sensitized preneoplastic cells to DNA-damage-induced cell death, and impaired skin tumorigenesis [100].

NEAT1 was found to be significantly upregulated in primary MM cells, with respect to its normal counterpart [104], and its knockdown antagonized MM cell growth both in vitro and in vivo, highlighting that NEAT1 depletion affects the HR repair pathway [93]. This finding is of relevance because it is an accepted notion that this pathway is deregulated in MM, contributing to GI, disease progression, and drug resistance [32]. Specifically, NEAT1 silencing leads to a significant downregulation of the phosphorylated RPA32 protein and RAD51B/1D transcripts [93]. RPA32 belongs to the replication protein A (RPA) complex, a heterotrimeric ssDNA binding structure essential in the HR-mediated repair. RPA complex binds to ssDNA at stalled forks and primes the HR repair cascade with the phosphorylation of the RPA32 subunit at specific sites by DNA-PK, ATM, and ATR kinases [105]. It has been shown that, at the DNA damage site, phosphorylated RPA32 directly interacts with RAD51, also reported as downregulated upon NEAT1 silencing of MM cells [93]. It is known that RAD51 represents a second key responder in HR-mediated repair and that cells deficient in RAD51 accumulate DSBs after replication or at stalled replication forks [106].

Furthermore, Adriaens et al. demonstrated that NEAT1 promotes ATR signaling in response to replication stress and that it is engaged in a negative feedback loop that attenuates oncogene-dependent activation of p53 [100].

Overall, these evidences underscore the involvement of NEAT1 in the regulation of the HR repair pathway in MM.

**Table 2:** ncRNAs regulating GI in MM.

miRNA lncRNA	Chromosome Location	Target/Pathway	Expression	Phenotypic Effects	Reference
**miR-29b**	Chr 7 (7q32.3)	Pro-inflammatory pathways (STAT3, NF-kB, cytokine/chemokine pathways)	Downregulated in MM PCs and DCs	Inhibition of MM proliferation; inhibition of osteoclast differentiation and activity; Inhibition of DC-induced polarization of Th cells into Th17 cells	[63,65,66]
**miR-22**	Chr 17 (17p13.3)	LIG3; ALT-NHEJ	Downregulated in MM PCs	Inhibition of MM growth in vitro and in vivo; Decrease of Bortezomib resistance	[26]
**miR-137**	Chr 1 (1p21.3)	AURKA	Downregulated by promoter CpG methylation	In vitro and in vivo anti-MM activity; increased bortezomib sensitivity	[72]
**miR-520g/miR-520h**	Chr 19 (19q13.42)	APE1	Downregulated in drug resistant MM cells	Inhibition of MM growth in vitro and in vivo	[73]
**miR-17-92**	Chr 13 (13q31.3)	BIM; NKG2D ligands	Upregulated in MM PCs	In vitro and in vivo promotion of MM growth; inhibition of trabectedin effects	[21,80]
MALAT1	Chr 11 (11q13.1)	Scaffold of PARP1-LIG3 complex that activates the A-NHEJ pathway	Upregulated in MM and plasma cell leukemias	Promotion of MM progression and drug resistance	[95,96]
NEAT1	Chr 11 (11q13.1)	RPA32; RAD51B and RAD51D (HR pathway)	Upregulated in MM	Promotion of HR pathway activity and drug resistance in MM cells	[93]

## 4. Future Perspectives

At present, it is acknowledged that the pathogenesis of MM depends on the acquisition of GI, which drives many features of malignant PCs, including dramatic genetic heterogeneity, proliferative advantage, and drug resistance.

Elevated GI represents a therapeutic vulnerability of MM PCs; accordingly, small molecule inhibitors targeting PARP or Aurora kinases, as well as spindle kinase inhibitors have been successfully tested in MM preclinical models and in early phase I/II trials; moreover, ATM, ATR kinase inhibitors, and DNA helicase inhibitors appear to be promising agents, displaying strong synergy in patients with highly refractory MM when combined with DNA-damaging agents, platinum derivatives, immunomodulators, and proteasome inhibitors [107].

In parallel, deregulation of the non-coding RNome has been recently regarded as a further mechanism prompting GI along MM onset and progression. Two major classes of non-coding RNA (ncRNA) molecules, i.e., miRNAs and lncRNAs, have been reported as crucial players in various GI cellular pathways, acting through the regulation of the transcription and/or the translation of GI machinery’s components. Indeed, exciting preclinical research has demonstrated that strategies aimed at the overexpression of tumor suppressor non-coding RNAs blocking relevant effectors of the GI pathways, or the inhibition of oncogenic non-coding RNAs affecting DDR responses, represent novel therapeutic weapons to antagonize ongoing GI. These studies provide the framework for potential clinical applications of ncRNA-based therapeutics to treat MM and other PC dyscrasias.

## Data Availability

Not applicable.
